# Lower Circulating Cytotoxic T-Cell Frequency and Higher Intragraft Granzyme-B Expression Are Associated with Inflammatory Interstitial Fibrosis and Tubular Atrophy in Renal Allograft Recipients

**DOI:** 10.3390/medicina59061175

**Published:** 2023-06-20

**Authors:** Brijesh Yadav, Narayan Prasad, Vinita Agrawal, Vikas Agarwal, Manoj Jain

**Affiliations:** 1 Department of Nephrology and Renal Transplantation, Sanjay Gandhi Postgraduate Institute of Medical Sciences, Lucknow 226014, India; brijeshbio146@gmail.com; 2 Department of Pathology, Sanjay Gandhi Postgraduate Institute of Medical Sciences, Lucknow 226014, India; vinita.agrawal15@gmail.com (V.A.); mnjjain@yahoo.com (M.J.); 3 Department of Clinical Immunology, Sanjay Gandhi Postgraduate Institute of Medical Sciences, Lucknow 226014, India; vikasagr@yahoo.com

**Keywords:** stable graft function, inflammatory interstitial fibrosis and tubular atrophy, cytotoxic T lymphocyte, granzyme B

## Abstract

*Background and Objectives:* Inflammatory interstitial fibrosis and tubular atrophy (i-IFTA) is an inflammation in the area of tubular atrophy and fibrosis. i-IFTA is poorly associated with graft outcome and associated with infiltration of inflammatory mononuclear cells. A cytotoxic T cell is a granzyme B^+^CD8^+^CD3^+^ T cell, mainly secret granzyme B. Granzyme B is a serine protease that may mediate allograft injury and inflammatory interstitial fibrosis and tubular atrophy (i-IFTA). However, there is no report identifying the association of granzyme B with i-IFTA after a long post-transplant interval. *Material and Methods:* In this study, we have measured the cytotoxic T-cell frequency with flow cytometry, serum and PBMCs culture supernatants granzyme-B levels with ELISA and intragraft granzyme-B mRNA transcript expression with the RT-PCR in RTRs in 30 patients with biopsy-proven i-IFTA and 10 patients with stable graft function. *Result:* The frequency of cytotoxic T cells (CD3^+^CD8^+^ granzyme B^+^) in SGF vs. i-IFTA was (27.96 ± 4.86 vs. 23.19 ± 3.85%, *p* = 0.011), the serum granzyme-B level was (100.82 ± 22.41 vs. 130.32 ± 46.60, *p* = 0.038 pg/mL) and the intragraft granzyme-B mRNA transcript expression was (1.01 ± 0.048 vs. 2.10 ± 1.02, *p* < 0.001 fold). The frequency of CD3^+^ T cells in SGF vs. i-IFTA was (66.08 ± 6.8 vs. 65.18 ± 9.35%; *p* = 0.68) and that of CD3^+^CD8^+^ T cells was (37.29 ± 4.11 vs. 34.68 ± 5.43%; *p* = 0.28), which were similar between the 2 groups. CTLc frequency was negatively correlated with urine proteinuria (r = −0.51, *p* < 0.001), serum creatinine (r = −0.28, *p* = 0.007) and eGFR (r = −0.28, *p* = 0.037). Similarly, the PBMC culture supernatants granzyme-B level was negatively correlated with urine proteinuria (r = −0.37, *p* < 0.001) and serum creatinine (r = −0.31, *p* = 0.002), while the serum granzyme-B level (r = 0.343, *p* = 0.001) and intragraft granzyme-B mRNA transcript expression (r = 0.38, *p* < 0.001) were positively correlated with proteinuria. *Conclusions:* A decrease in the CTLc frequency in circulation and an increased serum granzyme-B level and intragraft granzyme-B mRNA expression shows that cytotoxic T cells may mediate the allograft injury in RTRs with i-IFTA by releasing granzyme B in serum and intragraft tissue.

## 1. Introduction

Inflammatory interstitial fibrosis (IF) and tubular atrophy (TA) is an inflammation in the area of tubular atrophy and fibrosis [1]. Inflammation is an ongoing active event mediated by the inflammatory cell [2]. It is preceded by the terminal event of fibrosis. Inflammation and atrophy in the region of fibrosis and atrophy are strongly correlated with each other and are associated with poor graft survival [3]. Protocol renal allograft biopsy analysis has shown that mononuclear cell infiltration is correlated with chronic interstitial fibrosis and inflammation in the area of IF and TA. This could also predict the tubule-interstitial injury in subsequent biopsy [4]. Mengel et al. has reported that total inflammation scores, including inflammation in scarred and non-scarred areas, can predict the subsequent graft deterioration better than the inflammation scores only in viable areas [5]. Previous studies have also shown an infiltration of T-bet-positive mononuclear cells adjacent to the fibrotic area of the graft. This infiltration was also associated with inflammation [6,7]. However, the functional significance of T cells in inflammatory interstitial fibrosis and tubular atrophy has not been established yet. Infiltration of other cells, such as Treg, Th17 and Macrophage, is well appreciated, and these all are strongly associated with poor graft outcome [7]. Yet, the Treg cell is an anti-inflammatory subset and usually is converted into the inflammatory Th17 cell subset under the circumstance of the inflammatory cytokine milieu and fails to prevent allograft rejection [7]. For the appropriate function and survival of the Treg cell, the presence of an IL-2 cytokine is crucial. Treg cells utilize the majority of IL-2 and deprive the rest of the T-lymphocyte subset, resulting in their apoptosis [8]. IL-2 is also crucial for memory CD8+ T-cell formation [9]. An inhibition of IL-2 receptors by basiliximab is used to prevent acute allograft rejection [8]. The macrophage is another important inflammatory mononuclear cell that has been reported to be involved in renal allograft rejection by secreting inflammatory cytokines and chemokines and recruiting other inflammatory cells at allografts [6,7].

The cytotoxic T cell is a CD3^+^CD8^+^ granzyme-B^+^ cell, which secretes the cytolytic molecule granzyme B for killing target cells [10]. Granzyme B is a serine protease enzyme that is extracellularly secreted by activated cytotoxic T cells, NK cells, B cells and Treg cells [11]. Granzyme B activates several pro-proteins, such as IL-1β, IL-18, TGF-β and matrix-metallo-proteases, into their active forms, which mediates numerous inflammatory, cytolytic functions, contributing to inflammation and apoptosis in the target cell non-specifically; thus, circulating granzyme B may systematically affect different organs [12]. Therefore, we hypothesized that i-IFTA may be preceded by the inflammatory activity of cytotoxic T cells, which has been not studied in previous studies.

The inflammatory interstitial fibrosis–tubular atrophy may couple to the injury associated with granzyme B in the intragraft tissue compartment [7]. Studies have reported increased circulatory, intragraft and urinary levels of granzyme B, which could predict acute rejection with high specificity and sensitivity [13,14]. Nonetheless, there is little evidence on the role of cytotoxic T cells in the renal allograft recipient in the long-term post-transplant graft function [7,15] and no reports showing the role of cytotoxic T cells in inflammatory interstitial fibrosis and tubular atrophy. Therefore, the purpose of the current study is to profile the cytotoxic T cells in patients with biopsy-proven i-IFTA in the circulatory and intragraft compartments.

## 2. Materials and Methods

### 2.1. Patient Recruitment

In this study, a total of 40 live-related renal allograft recipients, who were admitted in the ward due to allograft dysfunction, were recruited. All patients underwent a routine biopsy procedure for the definitive diagnosis of renal allograft dysfunction. All patients were informed about the study through a patient information document, and a written consent form was obtained from each patient before the collection of blood and biopsy samples for analysis. Patients with stable graft function voluntarily participated in the study and gave consent to be included in the study and sample analysis for study purposes only.

### 2.2. Differential Diagnosis of Allograft Dysfunction

The histopathology reports of all the patients included in the study were independently assessed by two pathologists according to the Banff 2015 classification criteria [1]. Based on the histopathology reports, patients were assigned to either the stable graft function or the inflammatory interstitial fibrosis and tubular atrophy category (Table 1). A total of 30 patients were assigned to the i-IFTA group, and 10 patients were assigned to the stable graft function group.

### 2.3. Definition of Differential Diagnosis

Stable graft function was determined as a clinically <25% increase in serum creatinine levels from the baseline, which remained unchanged over the last 6-month period; nil proteinuria and a <10% cortical surface area showing the evidence of tubular atrophy, interstitial fibrosis and any glomerular or parenchymal changes; absence of C4d staining; and donor-specific antibodies in serum, as published in previous studies [6,15].

i-IFTA was defined as inflammation in interstitial fibrosis and tubular atrophy regions, atrophy in tubules [1,5]. Histopathology reports showing evidence of CNI toxicity, C4d^+^, cell-mediated rejection, viral-associated cytopathic changes, neutrophil pyelonephritis, de novo or persistent glomerulonephritis, or acute or chronic antibody-associated rejection were omitted from the analysis group.

### 2.4. Ethical Clearance

This research study was approved by the Committee on Institutional Ethics as per the Helsinki criteria declaration for subject recruitment in medical research. The code of ethics approval was IEC.2012-117-PhD-63.

### 2.5. Sample Collection

Blood and allograft biopsy samples were collected from each patient immediately after the biopsy procedure. Blood samples were obtained in heparinized vials and plain vials. The heparinized blood was used for the cytotoxic T-cell frequency measurement by flow cytometry and PBMCs separation for cell intact granzyme-B analysis. The blood sample of plain vials was used for the serum separation for the granzyme-B analysis by ELISA. One core biopsy specimen was collected in Trizole reagent (Thermo Fisher Scientific, Carlsbad, CA, USA) and immediately snap frozen in liquid nitrogen (LN_2_) until RNA isolation for the granzyme-B gene mRNA transcript expression analysis.

### 2.6. Cytotoxic T-Cell Staining and Frequency Analysis

#### 2.6.1. Cell Stimulation

One ml of heparinized blood was diluted in a 1:1 ratio with the complete RPMI 1640 (Sigma Aldrich, St. Louis, MO, USA) culture media, supplemented with 10% FBS and stimulated with the stimulants Phorbol 12-myristate 13-acetate (20 ng/mL; Sigma Aldrich, St. Louis, MO, USA) and Ionomycin (1 μg/mL; Sigma Aldrich, St. Louis, MO, USA) for 5 h. During the last 2 h of stimulation, 2 μM of Monensin (BD Biosciences, San Diego, CA, USA) was added as a protein transport inhibitor due to the stimulation. Cells were incubated in a cell culture incubator at 37 °C, 5% CO_2_ and 95% humidity for 5 h.

#### 2.6.2. Cytotoxic T-Cell Staining

A total of 100 μL of stimulated whole blood was used for the surface and intracellular staining. Surface staining was performed with 5 μL of Peridinin chlorophyll cyanine5.5 (Percp-Cy5.5) conjugated mouse monoclonal antihuman-CD3 and 10 μL of Allophycocyanin (APC) conjugated mouse monoclonal anti-human-CD8 antibody, following incubation at room temperature (RT) for 30 min in the dark. After washing with 5 mL PBS, the RBCs were lysed with RBC lysis buffer for 13 min, and the cells were washed and resuspended in cytofix/cytoperm solution for 20 min at RT. The cells were washed two times with PBS. Following fixation, cells were permeabilized with perm wash buffer (BD, Biosciences, USA), and intracellular staining with 5 μL of Phycoerythrin (PE) conjugated mouse monoclonal anti-human granzyme B was performed by incubating cells for 30 min in the dark at RT. For CD8 and granzyme B, an appropriate fluorochrome-conjugated isotype antibody was used to nullify any false positivity. All reagents and antibodies were purchased from BD Bioscience (BD Pharmingen, San Diego, CA, USA).

#### 2.6.3. Cell Analysis and Gating Strategy

A total of 10,000 lymphocyte gated cells were immediately acquired by a Facs caliber machine. Cells were analyzed by using FCS express software research edition version 7.18 (Denovo Software, Pasadena, CA, USA). The gating approach was first lymphocyte cells were gated in FSC-H and SSC-H plots, then CD3^+^ T cells were gated in lymphocyte cells. In the CD3 gate, CD8^+^ granzyme-B^+^ (double-positive cells in right top quadrant) cells were considered to be cytotoxic T cells. In the CD3 gate, CD3^+^CD8^+^ T cells were considered as CD8^+^ T cells.

### 2.7. Peripheral Blood Mononuclear Cell Intact Granzyme-B Level Measurement

PBMCs were separated from the heparinized blood following the protocol optimized in our laboratory. For the cell intact granzyme-B secretion assay, 1 × 10^6^ PBMCs/mL were cultured in 6-well flat-bottom culture plates in triplicate for 24 h by incubation in a cell culture incubator supplemented with 5% CO_2_ at 95% humidity and 37 °C. PBMCs were stimulated with non-specific mitogen stimulators, phorbol 12-myristate 13-acetate (20 ng/mL; Sigma Aldrich, St. Louis, MO, USA) and ionomycin (1 μg/mL; Sigma Aldrich, St. Louis, MO, USA). After 24 h of culture, supernatants were harvested by centrifugation and stored at −80 °C for granzyme-B level analysis in culture supernatants by ELISA.

#### 2.7.1. Serum Granzyme-B Level Analysis

Blood in plain vials was centrifuged at 1500 RPM for 5 min, and serum was extracted and stored at −80 °C until ELISA was performed with the BioLEGEND MAX^TM^ Human Granzyme-B ELISA (Bio legend, San Diego, CA, USA) Kit. ELISA was performed following the instructions mentioned in the datasheet of the Kit. In brief, pre-coated ELISA wells were washed 3 times with wash buffer, and 50 µL of assay buffer was loaded, followed by 50 µL of standard and serum samples loading in respective wells and incubation at RT for 2 h. The wells were washed 4 times, and wells were loaded with granzyme-B detection antibody and incubated for 1 h on a shaker. The wells were washed 4 times, and 100 µL of Avidin-HRP solution was loaded and incubated for 30 min on a shaker. The wells were further washed 5 times and loaded with 100 µL of substrate solution and incubated for 30 min in the dark. A total of 100 µL of stop solution was added to each well, and the reaction was terminated. The optical density of colored solution was read at 450 nm. The granzyme-B concentration of each patient was calculated by generating the Y = MX + C equation through a 4-P plot, with respect to the standard OD and respective concentration. The minimum detection limit of the kit for granzyme B was 2.4 ± 1.2 pg/mL.

#### 2.7.2. Intragraft Granzyme-B Gene mRNA Transcript Expression Analysis

RNA isolation: A single core of biopsy tissue was homogenized using the polytron Rotar Stator (Kinematica, AG, Luzern, Switzerland) homogenizer, and the RNA was extracted using the Qiagen RNA extraction mini-kit (QIAGEN, Courtaboeuf, France), following the manufacturer’s procedure. The purity and concentration of RNA were measured by the Nanodrop (Thermo Fisher Scientific, USA). RNA integrity was tested by electrophoresis on 1% of agarose gel.

Complementary DNA synthesis: Complementary DNA was prepared by using 500 ng of RNA and Random hexamer primer with SuperScript^TM^ II Reverse Transcriptase (RT) enzymes (Invitrogen, California, USA), following the manufacturer’s protocol, in a 20 μL reaction volume.

#### 2.7.3. Granzyme-B Gene mRNA Transcript Expression Analysis

Granzyme-B expression analysis was performed with the Taqman real-time PCR with 2 µL of cDNA in a 20 μL reaction volume using the Applied Biosystems GenAmp 7700 sequence detection system (Applied Biosystems, Foster City, CA, USA) and by using the Applied Biosystems pre-designed granzyme-B primer and probe (HS01554355_m1). Glucose-6 phosphate dehydrogenase (HS99999905_m1) was used as an endogenous housekeeping control to normalize the starting amount of RNA. The RT-PCR cycle was an initial denaturation of 10 min at 95 °C, followed by an alternate 40 cycles of denaturation at 95 °C for 15 s and amplification at 60 °C for 60 s. The relative fold change expression was calculated by using 2^−ddCt^ methods [16].

### 2.8. Statistical Data Analysis

Data were analyzed with SPSS software (IBM Corporation, Armonk, NY, USA), and graphs were plotted with Graphpad-8 software for Windows (GraphPad Software, La Jolla, CA, USA.). Data normality distribution was analyzed by the Shapiro–Wilk test. The continuous non-parametric variables were compared by using the Mann–Whitney test, and categorical variables were analyzed with the Chi-Square test by using Fisher’s exact test wherever needed. The parametric variables were analyzed with the independent samples *t*-test. Person correlations between CTLc profiles and the renal function parameter were performed. Values were presented in mean ± SD form. A *p* value < 0.05 for the two-tailed test was considered to be significant.

## 3. Results

### 3.1. Demographic and Clinical Profiles of Patients

The baseline demographic and clinical profiles of patients in both of the groups were similar, except for eGFR and proteinuria hemoglobin levels, which were significantly lower in the i-IFTA group, while serum creatinine and proteinuria were significantly higher in the i-IFTA group. The post-transplant interval of biopsy was 46.70 ± 17.30 months in the SGF group vs. 56.90 ± 25.37 months in the i-IFTA group. All patients received basiliximab as induction therapy. All patient were maintained on triple-immunosuppression Tacrolimus, Mycophenolate mofetil and Prednisolone (Table 2).

### 3.2. Circulating Cytotoxic T-Cell Frequency Was Lower in i-IFTA Patients

**Figure 1:**
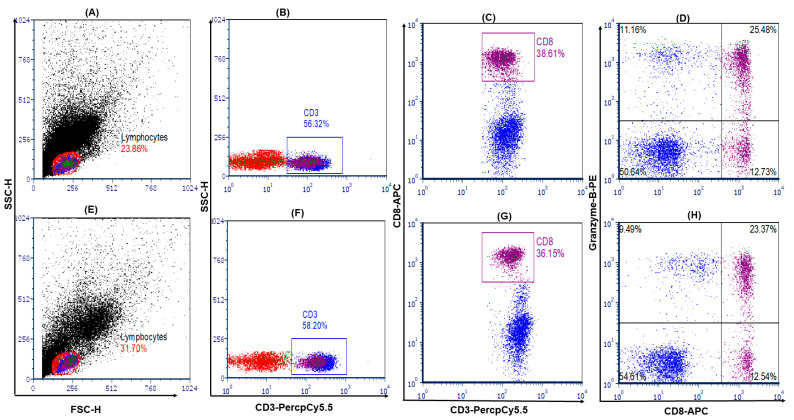
Flow cytometry image of cytotoxic T cells. Upper panel stable graft function and lower panel (**A**–**D**). Lower panel i-IFTA (**E**–**H**). First lymphocyte cells were gated in FSC-H and SSC-H plots, then CD3^+^ T cells were gated in lymphocytes cells. In CD3 gate, CD8^+^ granzyme-B^+^ (double-positive cells in right top quadrate) cells were considered to be cytotoxic T cells. In CD3 gate, CD3^+^CD8^+^ T cells were considered to be CD8^+^ T cells.

To determine the frequency of circulating granzyme-B^+^ cytotoxic T cells, we measured them with flow cytometry and found that the CD3^+^ T- and CD8^+^ T-cell frequencies were similar between the i-IFTA and SGF groups, while the CTLc (CD3^+^CD8^+^ granzyme B^+^) frequency was significantly lower in the i-IFTA group, suggesting granzyme-B discharge in serum by the activated CTLc (Table 3) and representative flow cytometry (Figure 1A–H).

### 3.3. Serum Soluble and Cell Intact Granzyme-B Level

**Figure 2:**
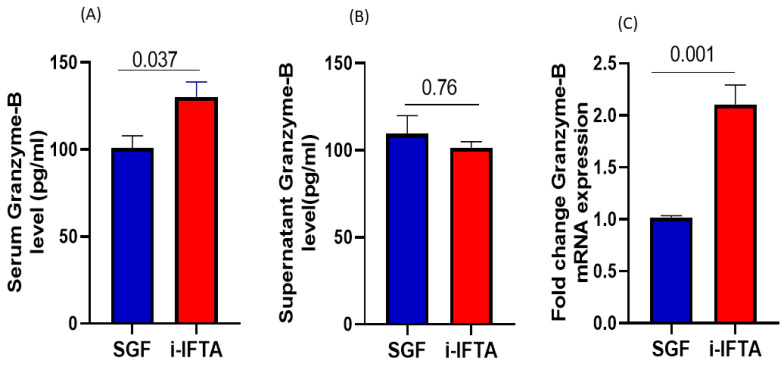
(**A**,**B**) Granzyme-B level was measured with the enzyme linked immunosorbent assay in serum and PBMCs culture supernatants. (**C**) Intragraft granzyme-B mRNA expression was analyzed with real-time PCR.

We measured the serum soluble and cell intact granzyme-B levels in serum and PBMC culture supernatants with ELISA. We found that the serum granzyme-B level was significantly higher in the i-IFTA group compared to SGF, whereas the PBMCs culture supernatants granzyme-B level was lower in the i-IFTA group, though there was no statistical difference between the SGF and i-IFTA groups (Figure 2A,B)

### 3.4. Intragraft Granzyme-B Gene mRNA Transcript Expression Analysis

To determine whether granzyme B expressed cell infiltration in intragraft tissue, we analyzed granzyme-B gene mRNA transcript expression with the Taqman RT-PCR. The expression level was significantly higher in the i-IFTA group compared to that of SGF, suggesting an infiltration of CTLc from the circulation to the intragraft compartment (Figure 2C).

### 3.5. Cytotoxic T-Cell Profiles Correlation with Kidney Function Markers

The CTLc frequency and PBMCs culture supernatants granzyme-B level were negatively correlated with the urine proteinuria and serum creatinine, while the serum granzyme-B level and intragraft granzyme-B gene mRNA transcript expression were positively correlated with proteinuria (Table 4).

## 4. Discussion

In this study, we have found that the frequency of circulating granzyme-B^+^ cytotoxic T cells was significantly lower and the intragraft granzyme-B mRNA transcript expression was significantly higher in the i-IFTA group. However, the CD3^+^ T- and CD8^+^ T-cell counts were similar between the groups. In addition, serum levels of granzyme B were significantly higher in i-IFTA patients, and the CTLc frequency and culture supernatant levels of granzyme B were negatively correlated with the proteinuria and serum creatinine, suggesting that persistent stimulation of cytotoxic T cells leads to increased synthesis and release of granzyme B in the circulation, as well as infiltration of granzyme B^+^ cells into the allograft tissue. An increased intragraft expression of granzyme-B mRNA has been reported to be associated with allograft injury in the late post-transplant period in patients with chronic antibody-mediated rejection [7,14,15]. A higher urinary granzyme-B mRNA expression level has been identified by many other studies as a non-invasive marker of acute rejection [17].

Granzyme B is a serine protease of cytolytic activity, expressed exclusively by the cytotoxic CD8^+^ T and NK cells. However, other non-cytotoxic cells, such as Tregs, basophils, monocytes, neutrophils and B cells, also secret extracellular granzyme B [18,19,20]. We found a similar level of CD8^+^ T cells in the circulation, although a higher granzyme-B level in serum, suggesting secretion of granzyme B by the activated CTLc. Further, in PBMCs culture supernatants, we noticed a similar level of granzyme B in the SGF and i-IFTA groups, suggesting a synthesis and intactness of granzyme B inside the cell, which may be released in serum after strong allogenic stimuli [7,10]. This further implies that even in patients with stable graft functioning, CTLc stores plenty of granzyme B, which may be released after receiving a specific stimulus. A similar level of granzyme B in culture supernatants between the SGF and i-IFTA groups further suggests the presence of some highly active subset of PBMCs that synthesize and release granzyme B after receiving a specific stimulus. The natural killer cell is another important cell, which is well known to secrete granzyme B and mediates renal allograft rejection [21].

In principle, intracellular granzyme B is directly delivered into the target cell by a perforin-dependent mechanism in a cell-to-cell contact-dependent fashion for inducing apoptosis in the engaged cell. However, the target of extracellular granzyme-B protease remains largely undetermined and acts non-specifically on different cellular molecules, resulting in the exacerbation of systemic inflammation usually experienced by organ transplant recipients [12,22]. In many other inflammatory diseases, such as rheumatoid arthritis, atherosclerosis and infections, an increased level of extracellular granzyme B has been observed, which was associated with inflammatory mediators and disease exacerbation [11,19,22]. We also observed that with an increase in the severity of Banff histological injury grades, the granzyme-B level was higher (Supplementary Table S1). It has been observed that extracellular granzyme B cleaves extracellular matrix (ECM) protein to induce tissue remodeling and cardiac fibrosis [12,23]. Granzyme B has been demonstrated to activate the pro-fibrotic molecule TGF-β. TGF-β favors the generation of both Treg and Th17 cells and the SMAD-dependent renal fibrosis pathway [12,24]. Cytokine IL-17 of Th17 cells is reported to induce SMAD/ERK-dependent signaling in renal fibrosis [25], although we did not analyze the SMAD-dependent fibrosis pathway. It would be interesting to determine whether granzyme B drives TGF-β-dependent fibrosis in transplanted grafts. One study showed that increased infiltration of Treg cells was associated with renal allograft rejection [7]. An insufficient number of Treg cells or defective Treg activity may be attributed to inflammatory cell response [26]. In addition, TGF-β is a profibrotic molecule that mediates the SMAD-3-dependent canonical pathway of renal fibrosis [27]. The inflammatory cytokines work beyond the infiltration site of cells. Thus, cytotoxic T cells present in the non-scarred area may induce inflammation in the viable area and may mediate apoptosis in the parenchymal cell, causing atrophy and fibrosis. This is further corroborated by the study showing that a deficiency of granzyme B leads to reduced cardiac fibrosis and hypertrophy [23]. Further, extracellular granzyme B has been shown to cleave the junctional protein vascular endothelial cadherin, accompanied by increased cell infiltration in the extracellular matrix, inflammatory cytokines and fibrotic gene expression in cardiomyocytes [23]. An increased intragraft granzyme-B^+^ mRNA transcript in our findings suggests an infiltration of granzyme-B-containing cells and the association of granzyme-B-mediated microvascular injury in allograft tissue (see Supplementary Table S1). Extracellular granzyme B also transforms pro-IL1 into active IL-1α, which is a potent inflammatory cytokine and fibrotic in nature [28], and may further increase the inflammatory cascade by the IL-1α/IL-1R signaling axis. IL-1α stimulates fibroblast cells to secrete interstitial collagen and extracellular matrix for tissue remodeling [29]. In vitro studies have shown that renal fibrotic-tissue-derived fibroblast cells were not inhibited by the IL-1 receptor blocker [30], suggesting the involvement of other fibrotic molecules, such as TGF-β. It has been reported that necrotic endothelial cells drive IL-1α to mediate chronic graft rejection [31]. Furthermore, inflammatory cytokines induce the expression of many other inflammatory cytokines, chemokines and receptors in the renal tubular epithelial cell, further encouraging the recruitment of other inflammatory cells and exacerbating the inflammation [6,7], which is further ratified by the observation that short-term high-dose steroid bolus therapy significantly reduces T-cell-mediated allograft rejection [32]. An increased intragraft and decreased peripheral blood granzyme-B mRNA have been reported to be associated with chronic antibody-mediated rejection, serving as a non-invasive rejection biomarker [7,15]. However, we found an increased serum level of granzyme B and increased granzyme-B mRNA in intragraft tissue, both suggesting granzyme-B synthesis, subsequent release and CTLc infiltration in the intragraft tissue. In addition, persistent allo-stimulation of cytotoxic T cells also leads to transient expression of different homing molecules, such as very late antigen activation-4 (VLA-4), antigen-associated leukocyte function (LFA-1), and CD103 and CXCR3 receptor chemokines, on their surface, which further help in binding to their cognate receptor (ICAM-1, MCP-1, MIP1-α) expressed on activated endothelial cells, resulting in endothelial cell injury [33,34].

To inhibit the destructive effect of granzyme B, immune cells naturally express granzyme-B inhibitors referred to as serine proteases inhibitors. It has been documented that in response to granzyme B, the tubular epithelial cells (TECs) express serine proteases-9, which prevents granzyme-B-mediated subclinical injury in the transplanted allograft and promotes graft survival [35]. Additionally, in response to viral dsRNA, epithelial cells express TLR3, which induces serin proteases inhibitor-9 expression in TECs to prevent CTLc-induced, virus-mediated graft damage [36,37], although we have excluded the patients with virus-associated histological changes from the study.

Additionally, CTLc expresses the surface molecule Fas-L, which mediates the FasL-Fas-dependent apoptosis in the renal tubular cell and serves as a marker for acute rejection [13,38]. Granzyme B also cleaves Bid, a proapoptotic molecule that binds to the Bax protein of mitochondria and forms a pore in the mitochondrial membrane, leading to the collapse of the membrane polarity, ROS formation and Cytochrome-c release in the cytoplasm to mediate apoptosis in the cell and ROS-dependent cell damage. Antioxidants, such as tocopherol and ascorbate, may neutralize the ROS and thus may delay granzyme-B-driven pathogenesis of inflammatory interstitial fibrosis and tubular atrophy.

Although our study is helpful in understanding the pathogenic mechanism mediated by the CTLc in i-IFTA patients, it has many limitations, too. In this study, we did not profile NK cells, a significant producer of granzyme B; similarly, we were unable to analyze Perforin and Fas-L expression in CTLc, pro-inflammatory cytokines TGF-β and IL-1β. However, the findings of this study provide deeper insight into the mechanism that may be used by CTLc to mediate graft injury and ultimately graft loss. This study highlights the pathogenic mechanism and how CTLc may mediate the pathogenesis of i-IFTA and allograft injury.

In conclusion, a decreased CTLc frequency in the circulation and an increased serum granzyme-B level and intragraft granzyme-B mRNA transcript expression may be associated with the pathogenesis of i-IFTA in RTRs.

## Figures and Tables

**Table 1:** Banff histological injury scores between SGF and i-IFTA patients.

Peritubular Capillaritis			
**Scores**	**SGF (*n* = 10)**	**i-IFTA (*n* = 30)**	***p***
p0	9 (90%)	25 (83.3%)	0.52
p1	1 (10%)	5 (16.6%)	
p2	0 (0%)	0 (0%)	
p3	0 (0%)	0 (0%)	
Glomerulitis			
g0	9 (90%)	23 (76.6%)	0.62
g1	1 (10%)	6 (20%)	
g2	0 (0%)	1 (3.3%)	
g3	0 (0%)	0 (0%)	
Tubulitis			
t0	9 (90%)	29 (96.6%)	0.44
t1	1 (10%)	1 (3.33%)	
t2	0 (0%)	0 (0%)	
t3	0 (0%)	0 (0%)	
Interstitial fibrosis			
i0	10 (14.2%)	0 (0%)	<0.001
i1	0 (0%)	6 (20%)	
i2	0 (0%)	18 (60%)	
i3	0 (0%)	6 (20%)	
Tubular atrophy			
t0	6 (60%)	2 (6.6%)	<0.001
t1	4 (40%)	7 (23.3%)	
t2	0 (0%)	15 (50%)	
t3	0 (0%)	6 (20%)	
Interstitial inflammation			
i0	8 (80%)	0 (0%)	0.002
i1	2 (20%)	18 (60%)	
i2	0 (0%)	9 (30%)	
i3	0 (0%)	3 (10%)	
Intimal arteritis			
v0	9 (90%)	11 (36.66%)	0.014
v1	1 (10%)	16 (53.3%)	
v2	0 (0%)	3 (10%)	
v3	0 (0%)	0 (0%)	
Interstitial fibrosis and tubular atrophy (IFTA)			
IFTA0	9 (90%)	0 (0%)	<0.001
IFTA1	1 (10%)	10 (33.3%)	
IFTA2	0 (0%)	15 (50%)	
IFTA3	0 (0%)	5 (16.7%)	

**Table 2:** Demographic and clinical characteristics of patients in SGF and i-IFTA.

Characteristics	SGF (Mean ± sd)	i-IFTA (Mean ± sd)	*p* Value
Pt. Gender (M:F)	10:0	22:8	0.068
Do. Gender (M:F)	2:8	8:22	0.673
Patient age (Years)	44.36 ± 8.20	40.88 ± 8.37	0.257
Post-transplant biopsy interval (Months)	46.70 ± 17.30	56.90 ± 25.37	0.246
eGFR (mL/min/1.73 m^2^)	70.62 ± 22.14	44.13 ± 13.83	<0.001
Tac level (ng/mL)	4.82 ± 0.98	5.02 ± 1.51	0.689
Hemoglobin (gm/dL)	12.94 ± 1.60	10.88 ± 1.61	0.001
TLC	8.39 ± 2.12	8.50 ± 5.02	0.94
BUN	25.79 ± 13.3	32.55 ± 8.84	0.074
Baseline creatinine (mg/dL)	0.81 ± 0.47	0.92 ± 0.41	0.500
S. Creatinine (mg/dL)	1.21 ± 0.18	2.22 ± 0.53	<0.001
24 h urine protein (gm)	0.16 ± 0.085	0.90 ± 0.61	<0.001
HLA mismatch	3.40 ± 0.69	3.10 ± 0.54	0.170
Induction regimen (Basiliximab)	10	30	1.00
Baseline Immunosuppression Tacrolimus + MMF + Pred	10	30	1.00
ESRD cause MN/HTN/NOS	6/3/1	20/9/1	0.69

**Table 3:** Cytotoxic T-cell frequency in SGF and i-IF-TA groups.

Characteristics	SGF	i-IF-TA	*p* Values
CD3^+^ T	66.08 ± 6.8	65.18 ± 9.35	0.68
CD3^+^CD8^+^ T	37.29 ± 4.11	34.68 ± 5.43	0.28
CD3^+^CD8^+^ granzyme-B T cell	27.96 ± 4.86	23.19 ± 3.85	0.011

**Table 4:** Correlation of CTLc frequency, serum and PBMC culture supernatants granzyme-B and intragraft granzyme-B expression with renal function parameters proteinuria, serum creatinine and eGFR.

	Proteinuria	Serum Creatinine	eGFR
CTLCs (%)	R = −0.51 *p* < 0.001	r = −0.28 *p* = 0.007	r = −0.28 *p* = 0.037
Serum Granzyme B (pg/mL)	r = 0.343 *p* = 0.001	r = 0.09 *p* = 0.3	r = −0.18 *p* = 0.09
Supernatants Granzyme B (pg/mL)	r = −0.37 *p* < 0.001	r = −0.31 *p* = 0.002	r = 0.27 *p* = 0.011
Fold change in intragraft mRNA	r = 0.38 *p* < 0.001	r = −0.12 *p* = 0.24	r = −0.061 *p* = 0.58

## Data Availability

Data related to the manuscript can be obtained from the corresponding authors upon reasonable request.

## Supplementary Materials

The following supporting information can be downloaded at: https://www.mdpi.com/article/10.3390/medicina59061175/s1. Table S1: Association of Intragraft Granzyme-B mRNA expression with Banff histological injury scores.

## Abbreviations

SGF—Stable graft function, i-IFTA—Inflammatory interstitial fibrosis and tubular atrophy, CTLc—Cytotoxic T lymphocyte cell, Gzm—Granzyme, RTRs—Renal transplant recipients.
