# Supplementary material for: Lower Circulating Cytotoxic T-Cell Frequency and Higher Intragraft Granzyme-B Expression Are Associated with Inflammatory Interstitial Fibrosis and Tubular Atrophy in Renal Allograft Recipients
Source: Medicina (Kaunas). 2023 Jun 20;59(6):1175. doi: 10.3390/medicina59061175 (PMC10305683; doi:10.3390/medicina59061175)
Supplement: Supplementary file 1 [file medicina-59-01175-s001.zip › medicina-2433996-supplementary.pdf]

Peritubular capillaritis		
Grade (number of patients)	Fold change (Mean+sd)	P-value
p0 (n=34)	1.73±0.82	0.13
p1 (6)	2.39±1.74	
**Glomerulonephritis**		
g0(32)	1.76±1.02	0.68
g1(7)	2.14±1.02	
g2(1)	1.87	
**Tubulitis**		
t0 (38)	1.85±1.02	0.56
t1 (2)	1.43±0.63	
**Interstitial fibrosis**		
i0 (10)	1.01±0.048	0.016
i1 (6)	2.41±0.95	
i2 (18)	1.98±1.18	
i3 (6)	2.16±0.51	
**Tubular atrophy**		
t0 (8)	1.50±1.12	0.16
t1 (11)	1.67±1.02	
t2 (15)	1.79±0.44	
t3 (6)	2.65±1.56	
**Interstitial inflammation**		
i0 (14)	1.55±0.93	0.20
i1 (26)	1.98±1.03	
**Intimal arteritis**		
v0 (20)	1.64±1.02	0.26
v1 (13)	1.83±0.47	
v2 (7)	2.37±1.52	
**Interstitial fibrosis and tubular atrophy (IFTA)**		
IFTA0 (9)	1.01±0.051	0.032
IFTA1 (11)	1.85±1.04	
IFTA2 (15)	2.20±1.02	
IFTA3 (5)	2.12±1.18	

Supplementary Table S1: Association of Intragraft Granzyme-B mRNA expression with Banff histological injury scores.
